# Acute effect of blueberry intake on vascular function in older subjects: Study protocol for a randomized, controlled, crossover trial

**DOI:** 10.1371/journal.pone.0275132

**Published:** 2022-12-01

**Authors:** Cristian Del Bo’, Massimiliano Tucci, Daniela Martini, Mirko Marino, Simona Bertoli, Alberto Battezzati, Marisa Porrini, Patrizia Riso

**Affiliations:** 1 Division of Human Nutrition, Department of Food, Environmental and Nutritional Sciences (DeFENS), Università degli Studi di Milano, Milano, Italy; 2 International Center for the Assessment of Nutritional Status (ICANS), Department of Food, Environmental and Nutritional Sciences (DeFENS), Università degli Studi di Milano, Milano, Italy; Kurume University School of Medicine, JAPAN

## Abstract

Aging is associated with an increased risk of developing cardiovascular disease which is often accompanied by a decline in vascular health and function. Current evidence suggests that berries may have a potential role in the modulation of vascular function, but dietary interventions are still needed to confirm findings, especially in older subjects. In the context of the MIND FoodS HUB project, this study aims to investigate the effect of a single serving of blueberry (250 g of blueberry versus a control product) in a group of older subjects (≥ 60*y*) through a randomized, controlled, cross-over dietary intervention trial. Specifically, the study evaluates the absorption kinetics of bioactives following the blueberries intake and the effects on markers related to oxidative stress, inflammation, and vascular function analyzed at different time points. By considering a drop-out rate estimate of 25%, at least 20 subjects will be recruited in the study. The study will provide evidence to support the potential beneficial effects of blueberry and its bioactive compounds on vascular function in a group of population more susceptible to vascular dysfunction and to the development of cardiovascular diseases. Moreover, the study will contribute the analysis of several metabolic and functional markers that can support the biological plausibility of the results obtained. Finally, the trial will provide data on the absorption and metabolism of blueberry bioactives which will be used to study their association with the different markers under study.

**Trail registration:** The trial is registered at ISRCTN (http://isrctn.com/ISRCTN18262533); May 7, 2021.

## Introduction

Aging is a complex and progressive phenomenon characterized by a decrease in functionality of numerous physiological processes at both molecular, cellular, tissue, and organ level, leading to an increased risk of oxidative stress, inflammation and, in turn, development of certain chronic degenerative diseases [1–5]. According to the Global Burden of Disease Study 2019, cardiovascular diseases (CVDs) still represent the leading cause of death in the world, particularly in older subjects [6]. The increased risk of developing CVDs during aging is mainly due to modifications to arteries and the establishment of vascular endothelial dysfunction [7]. The senescent process in endothelial cells leads to an alteration of arterial structure and functionality by reducing the production of vasodilators, such as nitric oxide (NO), and shifting toward the production of vasoconstrictors, procoagulants, proliferative and pro-inflammatory intermediates [8]. These factors are considered as critical for the development of endothelial dysfunction, arterial stiffness and vascular diseases [8–10].

Diet and dietary factors play a crucial role in maintaining normal physiological functions and promoting health. Among dietary factors, (poly)phenols, including flavonoids and phenolic acids, have been identified as potential bioactives able to improve CV health [11]. Recent observational studies report an overall inverse association between (poly)phenol intake and CV risk events and mortality [12–15]. In addition, mechanistic and human intervention studies seem to support the potential role of polyphenols in the modulation of several biomarkers related to vascular/endothelial function and CVDs [16–19]. Blueberries are one of the foods richest foods in (poly)phenols, particularly anthocyanins (ACNs), which are, among the (poly)phenols sub-classes, those mostly associated with CV health [19, 20]. In addition, blueberries are a source of minerals, vitamins, fiber, and salicylates [21] that could contribute, alone or synergistically, to the overall beneficial effect on vascular function [17, 22]. Short and long-term human intervention studies have documented that the consumption of blueberries may improve vascular function, reduce blood pressure and arterial stiffness in healthy subjects but also in those with risks for CVDs, metabolic syndrome, and type 2 diabetes [23–26]. To the best of our knowledge, few and contrasting studies have investigated the contribution of plant-based foods in the promotion of vascular function in older subjects [27] and only one trial tested the effect of blueberries, reporting no effect on markers of arterial stiffness [28]. Based on these premises, providing evidence of the potential health benefits of polyphenol-rich foods on vascular health is relevant in the current demographic context also due to the almost world-wide increase in the number of older people [29].

## Aim

The goal of this study is to evaluate the effect of a single portion of blueberries on markers of vascular function, oxidative stress, and inflammation in older subjects. In addition, the absorption kinetics of blueberries’ bioactive compounds will be assessed and the data obtained correlated with the other biological markers under study.

## Methods/Design

### Protocols and study design

**Fig 1:**
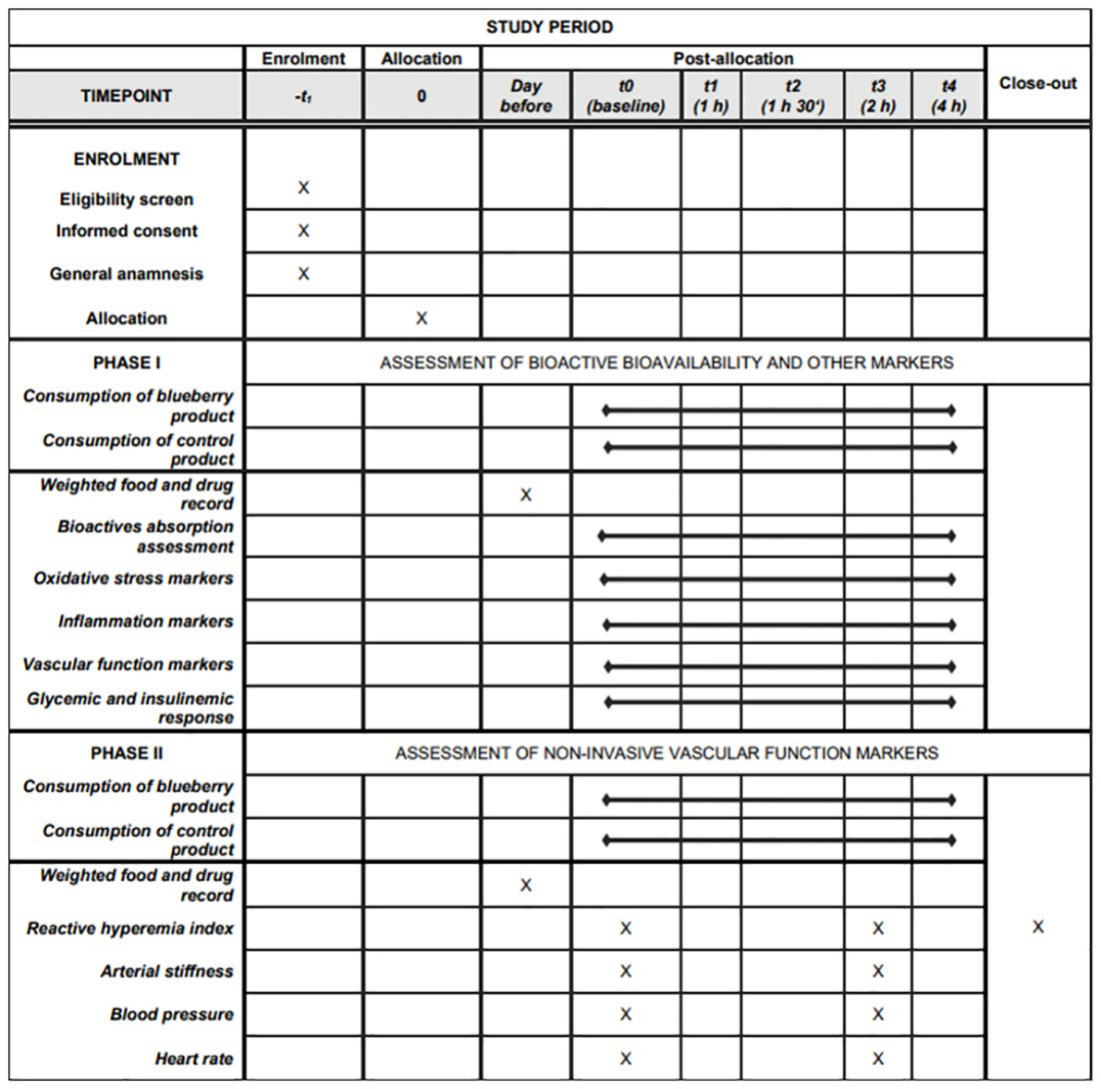
Schedule of enrollment, interventions, and assessment: Recommendations for interventional trials (SPIRIT).

This study follows a randomized, controlled, cross-over design (single portion of blueberry versus a control product) in a sample of free-living older subjects (≥ 60*y*). The study involves two phases (Phase 1 and 2), both including two different appointments, each one spaced by a 1 week wash-out period, as illustrated in the SPIRIT schedule of enrollment, interventions, and assessments (Fig 1 and S1 Checklist).

**Fig 2:**
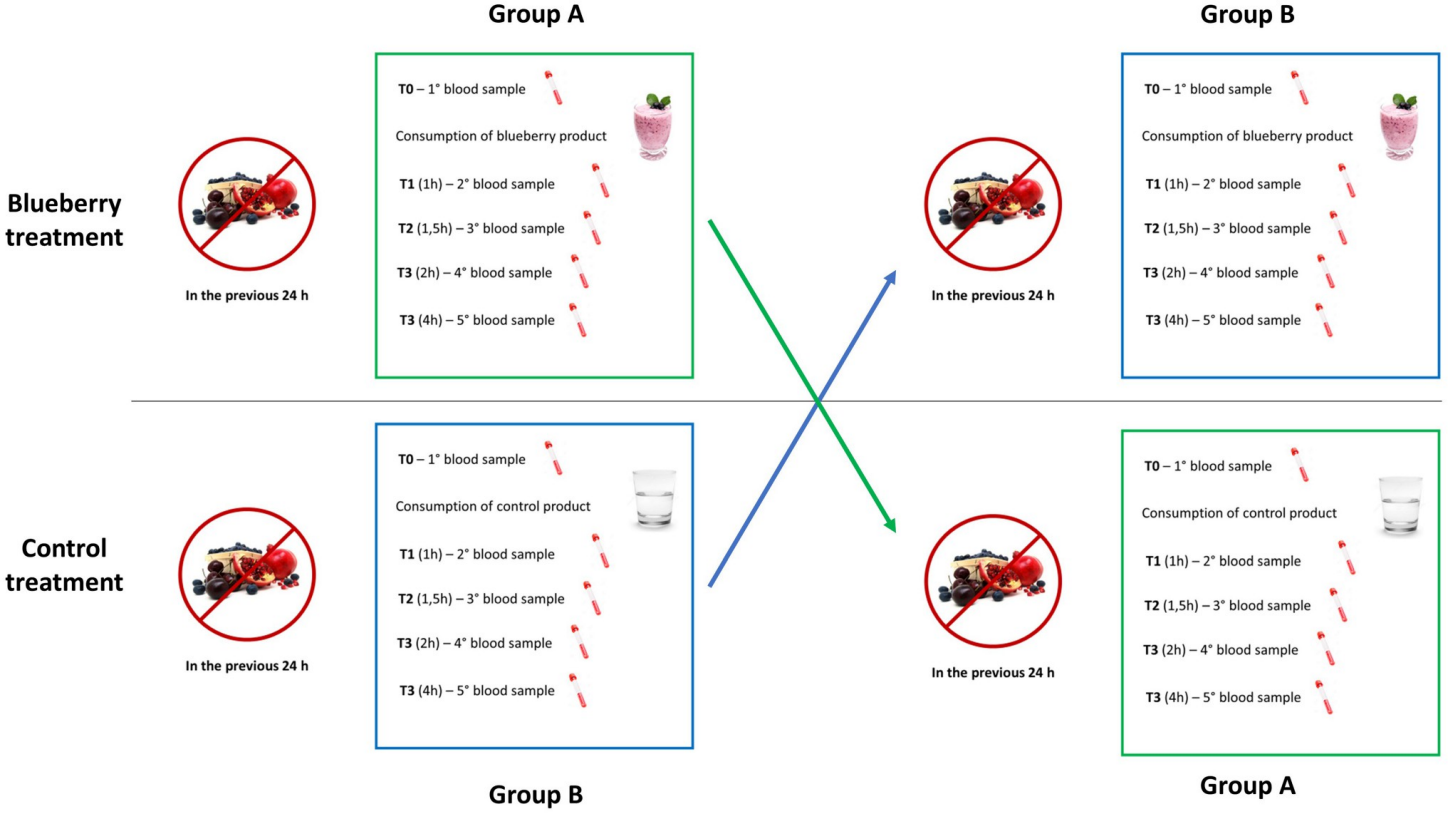
Schematic representation of the study design for the evaluation of blueberry bioactive absorption and of different metabolic/functional markers (phase 1).

**Fig 3:**
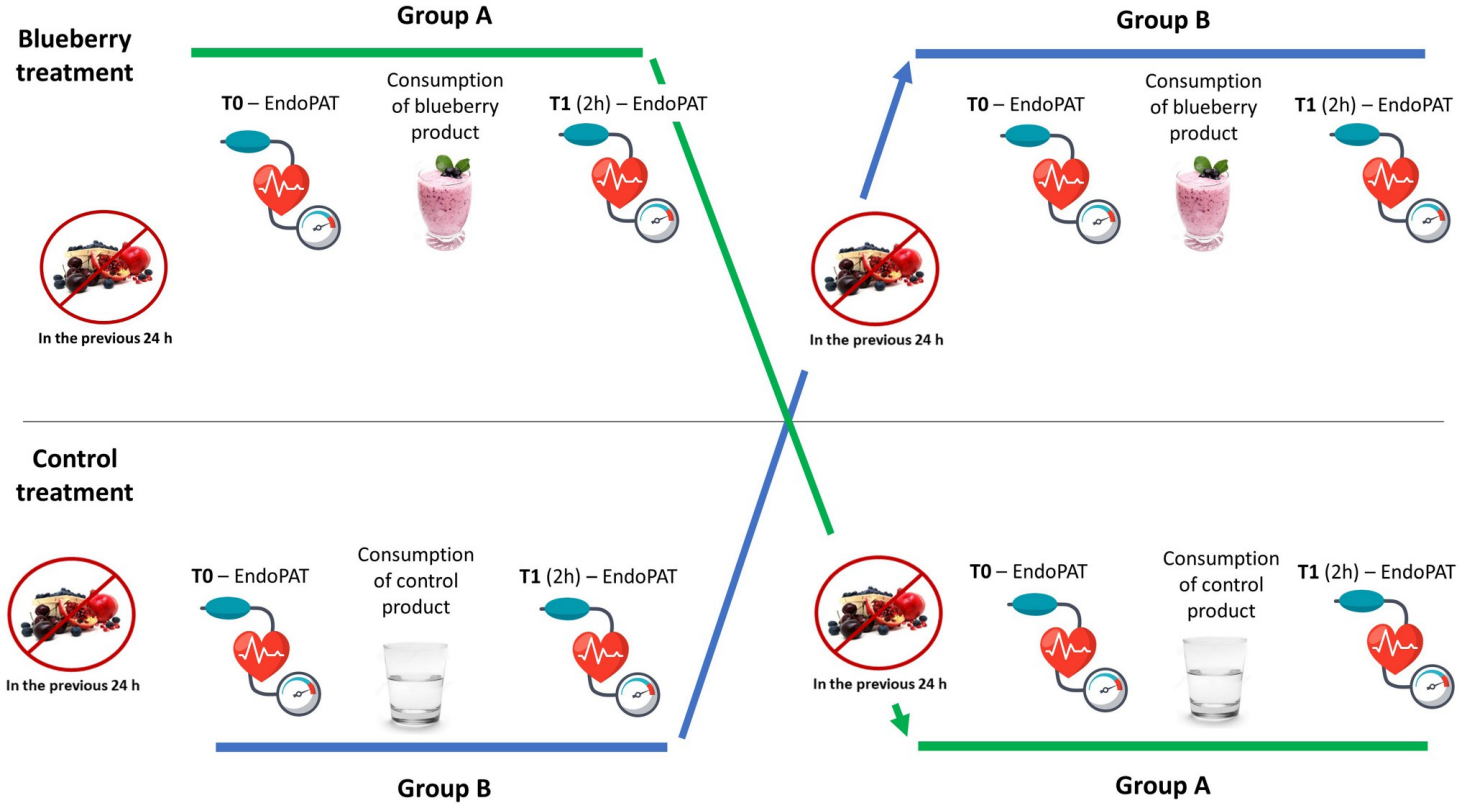
Schematic representation of the study design for the evaluation of the effects of intervention on vascular function markers (phase 2).

Phase 1 is aimed at evaluating the absorption of blueberry polyphenols and the effect of blueberries consumption on markers of vascular function (i.e., endothelin-1, NO, ICAM-1, VCAM-1 and VEGF), inflammation (i.e., IL-6, IL-8, and TNF-*α*), oxidative stress (i.e., levels of DNA damage), blood glucose and insulin response (Fig 2). Phase 2 is devoted to the evaluation of vascular functionality, using a non-invasive biosensor and the assessment of reactive hyperemia index (RHI), Framingham reactive hyperemia index (fRHI) and arterial stiffness (i.e., augmentation index (AI) and AI@75); in addition, blood pressure is recorded, as depicted in Fig 3. The approach, divided into two phases, is necessary to facilitate blood drawing (venous cannula) and the evaluation of vascular function (at the level of the brachial artery) avoiding potential sources of interferences between the two measurements. The day before the experiment, participants receive a list of instructions to follow. Following these instructions, subjects are asked to maintain their habitual lifestyle habits (also in terms of physical activities) and to refrain from consuming foods rich in (poly)phenols (e.g., berries, tea, coffee, chocolate, fruit juices). In addition, to limit the possible noise deriving from a long-fasting period the day of the experiment, subjects participating at the Phase 1 are invited to consume a standardized light breakfast. Specifically, volunteers can consume 200 mL of partially skimmed milk or 125 g of yogurt and 3 biscuits (i.e., shortbread) or 3 rusks. The meal has to be consumed at least 90 min before blood collection and at the same hour each test day. The adherence to instructions is evaluated through a food diary and a face-to-face interview that will be scheduled the day of the experiment. In addition, subjects are motivated to complete all the intervention by providing them with free meals the test days and with the access to all laboratory analysis at the end of the trial.

### Trial status

The trial has been prospectively registered (May 7, 2021; ISRCTN18262533) and is currently ongoing.

### Location

The study is carried out at the Department of Food, Environmental and Nutritional Sciences (DeFENS), Università degli Studi di Milano (Milan, Italy) through its International Center for the Assessment of Nutritional Status (ICANS).

### Participant enrollment

For the study, a group of 20 subjects (age ≥ 60*y*; 10 women and 10 men) are enrolled. The enrollment is carried out through advertisements by using emails, social networks, bulletin boards, and by word of mouth. The subjects who express their willingness to participate undergo a first general medical anamnesis through questions about their health status and their lifestyle (e.g., diseases, use of drugs and supplements, dietary habits, alcohol consumption, and physical activity) followed by an accurate clinical evaluation to confirm their eligibility to participate. Subjects interested in participating in the study sign an informed consent in which they agree with all the information on the dietary intervention, the analysis, and protocols that they are asked to follow. Volunteers are selected according to the inclusion and exclusion criteria reported below:

### Inclusion criteria

- Age ≥ 60*y*.

### Exclusion criteria

- Diabetes;
- Major condition related to cardiovascular system (e.g., a medical history of thrombosis or myocardial infarction);
- Presence of allergies (or other adverse reaction) to the ingestion of blueberry.

Changes in the volunteer status during the experimentation (e.g. occurrence of an exclusion criteria such as drug prescription, not evidenced at the enrolment) will be assessed to define the need of final exclusion from the study.

### Blueberry and control product

Blueberry product consists of a mousse obtained by shredding a serving of blueberries (organic highbush blueberries, cultivar Legacy) able to provide at least 300 mg of anthocyanins (ACNs). This amount of ACNs is considered sufficient to induce an improvement of vascular function as previously reported [30, 31]. The control product consists of a drink prepared by suspending the same amount of fructose, glucose, and saccharose of blueberry (matching the blueberry product for energy) in water, as already published [30, 31].

### Information on potential adverse effects

Even though no reports of adverse effects due to a blueberry consumption have been registered or reported in the literature, subjects are advised to annotate and communicate any adverse symptom perceived during the intervention period.

### Biological sampling

Blood samples (7 mL) are collected at baseline (t0) and after 1 h (t1), 1 h 30 ‘(t2), 2 h (t3) and 4 h (t4) from the consumption of blueberry or control product, as depicted in Fig 2. A cannula needle is used to facilitate blood collection procedures and minimize the discomfort of the volunteers. Tubes containing silicon for serum and tubes containing heparin as anticoagulants for peripheral blood mononuclear cells (PBMCs) are used. An aliquot of blood (500 μL) is immediately processed to obtain PBMCs, while the rest of the blood is maintained at room temperature (22°C) for 30 min before being processed by centrifugation at 1088 g for 15 min at 4°C. The serum obtained is collected, divided in aliquots, and stored at -80°C until analysis.

### Outcome measurements

The primary selected outcome of the study is RHI as a vascular function marker, while other markers including vascular function (i.e., fRHI, AI, AI@75, Endothelin-1, NO, ICAM-1, VCAM-1 and VEGF), inflammation (i.e., IL-6, IL-8, and TNF-*α*) and oxidative stress markers (DNA damage), together with circulating levels of (poly)phenols, salicylates, glycaemia and insulin, are included as secondary outcomes to support and validate the study hypothesis.

### Anthropometric measurements

Body weight and height are assessed at the beginning of the study by trained personnel following the international guidelines reported by Lohman et al. [32]. BMI calculation is obtained by dividing a person’s weight by the height to the power of 2 (BMI = weight (kg) / height (m)2).

### Blood pressure

Volunteers are monitored during each intervention period measuring both systolic and diastolic blood pressure (SBP and DBP) obtained in a resting, seated position based on the validated JNC 7 guidelines [33].

### Metabolic and functional markers

At enrollment, metabolic and functional parameters (i.e., glucose, insulin, lipid profile, liver, and renal function) are analyzed by a standardized validated protocol, using an automatic biochemical analyzer (YSI 2300 STAT Plus Glucose and Lactate Analyzer, Marshall Scientific, Hampton, VA, USA)). In details, non-high density lipoprotein cholesterol (non-HDL-C) is calculated by subtracting high density lipoprotein cholesterol (HDL-C) from total cholesterol (TC) while low density lipoprotein cholesterol (LDL-C) concentration is estimated using the Friedewald formula [34]. The levels of insulin sensitivity are assessed using the widely used homeostasis model assessment for insulin resistance (HOMA-IR), that is calculated considering fasting plasma concentrations of glucose and insulin, as indicated by Wallace et al. [35]. The blood samples are used for the evaluation of: i) kinetics of the absorption of the anthocyanins by gas chromatography–mass spectrometry analysis (GC–MS); ii) kinetics of the absorption of salicylates by means of ultra-performance liquid chromatography tandem mass spectrometry (UPLC-MS/MS); iii) serum markers of vascular function (i.e. ET-1, NO, ICAM-1, VCAM-1 and VEGF) and inflammation (i.e. IL-6, IL-8, and TNF-*α*) evaluated through ELISA kit, while serum markers of oxidative stress (i.e. levels of DNA damage) evaluated through comet assay iv) blood glucose concentrations evaluated by oxidation with YSI 2300 STAT Plus Glucose and Lactate Analyzer and insulin levels determined by ELISA kit.

### Vascular function and arterial stiffness

Endothelial-dependent vasodilation in the small finger arteries is assessed by a non-invasive plethysmographic method (Endo-PAT 2000, Itamar Medical Ltd, Caesarea, Israel) as described elsewhere [36, 37]. Briefly, the Endo-PAT equipment consists of two finger-mounted probes, which include a system of inflatable latex aircushions. The pulsatile volume changes of the fingertip are sensed by a pressure transducer, located at the end of each probe, and transferred to a personal computer where the signal is transduced, amplified, displayed, and stored. For the evaluation, subjects are requested to lie down in a supine position, with both hands on the same level, in a comfortable and thermoneutral environment. Then, a blood pressure cuff is placed on one upper arm (study arm), while the contralateral arm serves as a control arm. After a 10 min equilibration period, the blood pressure cuff on the study arm is inflated to 200–220 mmHg for 5 min. The cuff is then deflated to induce reactive hyperemia while the signals from both PAT channels (Probe 1 and Probe 2) are recorded by a computer. The RHI, an index of the endothelial-dependent flow-mediated dilation, is derived automatically in an operator independent manner. In fact, RHI is calculated as the ratio of the average pulse wave amplitude during hyperemia (60–120 s of the post-occlusion period) to the average pulse wave amplitude during baseline in the occluded hand, divided by the same values in the control hand and then multiplied by a baseline correction factor. According to manufacturing indications, an RHI value of 1.67 provides a sensitivity of 82% and a specificity of 77% for diagnosing endothelial dysfunction [38].

The Endo-PAT tool also provides the digital augmentation index (AI), a measure of pulse wave reflection and a surrogate marker for arterial stiffness. AI derives from digital pulse volume waveforms and it has been reported to be strongly correlated with aortic AI. Peripheral AI is calculated from the shape of the pulse wave recorded during baseline [39, 40]. Since digital AI is affected in an inverse and linear manner by heart rate (HR) [41], the AI was automatically normalized by considering a HR of 75 bpm (AI@75).

### Vascular and inflammatory markers

The concentrations of several markers related to vascular and inflammatory processes are quantified at least in duplicate using specific ELISA or colorimetric assay kits according to the instructions of each producer (Boster Bio, Valley Ave, Pleasanton, CA; Cayman Chemical Company, Ann Arbor, MI, USA). The following kits are used on serum samples obtained at each time points during the intervention periods: IL-6: BSR-EK0410—Human IL-6 PicoKine ELISA Kit, IL-8: BSR-EK0413—Human IL-8 PicoKine ELISA Kit, TNF-*α*: BSR-EK0525—Human TNF alpha PicoKine ELISA Kit, Endothelin-1: BSR-EK0945—Human Endothelin PicoKine ELISA Kit, ICAM-1: BSR-EK0370—Human ICAM-1 PicoKine ELISA Kit, VCAM-1: BSR-EK0537—Human VCAM-1 PicoKine ELISA Kit, VEGF: BSR-EK0539—Human VEGF PicoKine ELISA Kit, NO: CAY-780001–2x96—Nitrate/Nitrite Colorimetric Assay Kit.

### Oxidative stress markers

The levels of oxidatively-induced DNA damage, as markers of oxidative stress, are assessed in PBMCs by the comet assay on an aliquot of fresh blood, immediately after its withdrawal. Oxidatively-induced DNA damage is measured by treating the cells with hydrogen peroxide and by evaluating the capacity of cells to counteract this oxidative insult. This procedure is performed according to Del Bo’ et al., [42]. Briefly, cell resistance against oxidatively induced DNA damage is measured by treating the isolated PBMCs with hydrogen peroxide (H2O2). Two slides with two gels each are prepared for each sample. One slide is treated with H2O2 (500 μmol/L in PBS) for 5 min in the dark, while the other slide is treated with PBS without H2O2, as it is used as a control. Following oxidative treatment, the slides are placed in lysis buffer (2.5M NaCl, 0.1M Na2 EDTA, 10mM Tris, 1% Triton X-100, 1% DMSO, and 1% N-lauroylsarcosine sodium salt, pH 10) for 1h at 4°C in the dark. After lysis, the gels are washed in buffer (40nM HEPES, 0.1M KCl, 0.5mM EDTA and 0.2mg/ml bovine serum albumin, pH 8) and then transferred to electrophoresis buffer (0.3M NaOH and 1mM Na2 EDTA) and incubated for 40min at 4°C in the dark. Electrophoresis is performed at 1.1V/cm2 for 20min. Slides are successively neutralized (0.4M Tris–HCl, pH 7.5) for 15min at 4°C in the dark, stained with ethidium bromide (2 μg/ml), washed in PBS, drained and coverslipped to permit the subsequent quantification of DNA damage through an epifluorescence microscope attached to a high sensitivity CCD video camera and to a computer equipped with an image analysis system (QCapture 2.9.13, Irvine, CA, USA; DNA COMET1.50.00, University of Milan, Italy). The levels of DNA damage are calculated as the percentage of DNA in the tail.

### Serum anthocyanins

Serum samples are used to determine the kinetics of absorption of blueberry ACNs and salicylates. The extraction and analysis of ACNs from serum are performed through a microelution solid phase extraction (SPE) and ultra-HPLC methods, according to Martí et al., [43]. Briefly, prior to injection in the UPLC-MS/MS, ACNs are extracted using μElution Plates 30 mm conditioned with 250 mL of methanol and 250 mL/mL of 0.2%acetic acid. Then 350 μL of plasma is mixed with 350 μL of phosphoric acid 4%, loaded into the plate. The loaded plates are washed with 200 μL of Milli-Q water and 200 μL of 0.2%acetic acid. Then, the retained ACNs are eluted with 2 x 50 μL of acetone/Milli-Q water/acetic acid solution (70:29.5:0.5, v/v/v) and injected. Injection volume is 2.5 μL. UPLC-ESI-MS/MS is constituted by a stationary phase of 100% silica particles, particles while the mobile phase by 10% acetic acid (eluent A) and acetonitrile (eluent B). The flow rate is set at 0.4 mL/min.

### Blood salicylates

The analysis of salicylates in serum samples is performed using stable isotope dilution and gas chromatography–mass spectrometry analysis (GC–MS), as described previously [44]. Briefly, following adjustment for the pH to about 1.0 with hydrochloric acid, the organic material is extracted twice with ethyl ether and ethyl acetate (2 mL), and then is collected for derivatization prior to GC–MS analysis. Analysis is then carried out using a gas chromatograph interfaced with a single-quadrupole mass spectrometer equipped with a chamber provided with an electron ionization source and using helium as carrier gas. Finally, the identification of derivative salicylic acid is achieved by comparing the gas chromatographic retention times and the mass spectra of the serum samples with those of the standards.

### Data management

Data management includes a strict control of data quality and check of accuracy of data entry. To ensure data protection, data will be saved in a password-protected Excel file on an electronic device equipped with an antivirus system locked up in a secure location when not in use. Once completed final data will be stored in an electronic database (Dataverse). Identifiable data are not recorded in the database or other documents, and participants are identified by a unique trial ID only. Hard copies of data sheets linking the participant identification number to the person’s contact details are kept securely within the University premises that are accessible only to dedicated research team members. Participant files and other source data (including copies of protocols, questionnaires, and original reports of test results, correspondence, records of informed consent and other documents pertaining to the conduct of the study) are kept for the maximum period of time permitted by the institution.

### Sample size, randomization, and statistical analysis

The sample size is determined based on previous studies conducted in our laboratories (30–31) and other research groups [26, 28, 45] to test the efficacy of a blueberry consumption on markers of vascular function. Twenty volunteers are enrolled for the study. This number is considered sufficient (alpha = 0.05, 80% power) to determine an increase of 0.30 in the RHI parameter (endothelial function marker) after blueberry intake. This calculation also considers a possible drop out of 25% of the volunteers. Subjects are randomly divided using a computer random number generator. The randomisation and allocation is performed by a person not involved in the trial and blinded to the investigators and researchers involved in samples analysis. The statistical analysis is performed by means of STATISTICA software (Statsoft Inc., Tulsa, OK, US). Only complete data obtained will be analysed. The Shapiro–Wilk test is applied to verify the normal distribution of the variables under study. In particular, the following statistical elaborations are performed to identify significant differences between treatments: (i) the analysis of variance (ANOVA) with repeated measures, (ii) Wilcoxon paired data test, (iii) Linear Mixed Model (LMM) analysis. Furthermore, regression and correlation analyses (Spearman and Kendall test) are carried out to highlight potential associations between vascular activity, and markers of inflammation, vascular function, oxidative stress, other than biochemical and physiological markers. Corrections for potential confounding factors (i.e., age, gender, drugs/medications) are performed. When appropriate, a post-hoc p-value adjustment is performed using the Hochberg-Benjamin correction. Significance is set at p<0.05; significance in the range 0.05 < p < 0.10 is indicated as trend.

### Monitoring

Given the limited objectives and the short-term nature of the intervention, this trial is monitored by the dedicated research team without the use of a formal data monitoring committee and will be performed independently from the sponsor. Data access is restricted to trained staff with unique password protected accounts. Eventual adverse events such as unfavorable and unintended signs, abnormal laboratory findings, and symptoms temporarily associated with the intervention are collected from the time of intervention until the end, whether or not considered related to the intervention study. All adverse events are considered until they are resolved, but adverse effects are unlikely to occur.

### Ethics and dissemination

The study is conducted in accordance with the Declaration of Helsinki and the Data Protection Act. Trial personnel obtain informed consent from all participants prior to inclusion to the study, including consent for the use of data and biological specimens in other ancillary studies. The principal investigator stores informed consent. All subjects must agree to participate voluntarily and are free to withdraw from the study at any time. The study (S1 File) received ethical approval by the Ethics Committee of the University of Milan on the 12 December 2020 (S2 File). The Research Ethics Committee (REC) approval includes the trial protocol, information sheet and consent form. The trial is registered at the International Standard Randomized Controlled Trial Number (ISRCTN18262533, date of registration: 07 May 2021). Any amendment to the protocol and information provided to participants are submitted to the REC for approval prior to implementation. Substantial amendments may only be implemented after written Ethics Committees approval has been obtained, whereas non-substantial amendments can be implemented without written approval from the Ethics Committee. The Principal Investigator (PI) has to ensure that the participant’s privacy is maintained. Data and source documents are stored in such a way that they can be accessed later for the purposes of monitoring or inspection by the Ethics Committee. Public access to data and other materials could be requested to the PI at the end of the study after completion of elaboration and publication of data. At the end of the study, participants can receive a copy of the results of the study from the PI. The results from the trial are submitted for publication in a peer-reviewed journal irrespective of the outcome. Authorship of presentations and reports related to the study are in the name of the collaborative group and the list will be based on the extent of contribution. Results will be also communicated through other dissemination means including relevant scientific conferences.

## Discussion

The prevention of the decline of endothelial function, especially in older subjects, represents an important task in view of CV events prevention. In this context, diet and dietary factors can contribute not only to optimal aging, but they can also play an important role in the preservation of the vascular system. As reported above, diet is a well-known pillar of optimal aging; however, little evidence is available about the effect of bioactive rich-foods (such as polyphenol-rich foods) on vascular function in older subjects. Thus, there is a need for research that pushes towards acquiring solid evidence. The exploitation of well-controlled and specifically targeted dietary intervention trials represents the correct scientific approach for the evaluation of the health promoting properties of food bioactives, overcoming and completing the observations deriving from epidemiological studies and pre-clinical models. On the other hand, human interventions are difficult to implement in practice, as they can be affected by several confounding factors (e.g., environment, diet, lifestyle) and require considerable efforts in terms of organizational skills, in particular when they address specific target populations such as older subjects.

Previous human intervention studies conducted in our laboratories have documented that the intake of a single portion of blueberries (300 g, providing at about 300 mg of ACNs) counteracted (2 hours post consumption) an impairment in vascular function and blood pressure in a group of healthy young smokers with normal endothelial function [30]. In another study, we found that the same blueberry portion increased vascular function in both young smokers and non-smokers with endothelial dysfunction (2 hours from intake) [31]. Conversely, we have shown that a portion of blueberry purée (300 g) did not affect vascular reactivity (measured 1 hour after intake), but reduced oxidative stress, in a group of young healthy volunteers with normal vascular function [46]. Similarly, we could not demonstrate an effect on vascular function and inflammation, while there was a reduction in oxidative stress markers, after a 6-week intervention with a wild blueberry-based drink (250 mL/d, providing 475 mg of ACNs) in adults with CVD risk factors [47].

Within this study, we want to test the hypothesis that the consumption of blueberries can positively affect markers of vascular function in older subjects. Considering that aging is associated with an increased risk of oxidative stress and inflammation, and that these two conditions are crucial in the onset and progression of vascular dysfunction, we want also to ascertain whether the intake of a single blueberry portion can exert a positive modulation on a plethora of direct and indirect markers of oxidative stress and inflammation. Furthermore, since the potential health benefits should be attributed to the absorption of the bioactive compounds, the kinetic of absorption of (poly)phenols and salicylates from blueberries is evaluated. To this aim, a randomized, controlled, crossover post-prandial study is performed in which the absorption of bioactives from the intake of a single portion of blueberries and the effects on metabolic and functional markers (e.g., vascular function, oxidative stress, and inflammation) is evaluated. The exploitation of a crossover design allows us to reduce the interindividual variability of the subjects, with advantages in terms of sample size and control of the experimental plan for potential confounding factors. The intervention is planned at the facilities of the University in a controlled environment in which subjects can rest, comply with the instructions provided and consume the products. The products consist of a serving of blueberries and a control product. The blueberry portion is defined by considering the amount of ACNs present and potentially capable of exerting a beneficial effect, but also that the portion should be easily consumed as part of a balanced diet. Based on our previous studies, the portion should be in the range 250–300 g (raw product) able to provide at least 300 mg of ACNs [30, 31]. The control product consists in a sugar drink (250–300 mL) containing the same sugars and amount present in the blueberries, matching for macronutrients and energy intake. The inclusion of a control drink with these characteristics allows us to maintain a controlled experimental setting. Subjects also have to follow, 3-day before and during the experimentation, specific dietary instructions consisting of a list of foods allowed and not allowed. Furthermore, weighed food diaries are assessed by each volunteer both before and during the intervention to permit an accurate estimation of nutrient intake and to check the compliance with the dietary instructions by maintaining a high degree of control.

The study is performed in two different phases. Phase 1 consists in the evaluation of the absorption of blueberry bioactives, metabolic and functional markers analyzed in the blood samples collected at different time points (time zero (0 h; baseline), 1 h, 1 h 30 min., 2 h and 4 h from the consumption of blueberry or control products) selected by taking into consideration the “regular” pharmacokinetic of ACNs in terms of time of maximum plasma concentration (tmax; at 2 h), peak of maximum plasma concentration (Cmax) and clearance from blood. These times have been selected based on the results of previous studies showing the absorption of ACNs and a modulation of metabolic and functional markers, including oxidative stress and inflammation, in young healthy subjects or subjects with risk factors following the consumption of blueberries and/or blueberry-bioactives [45–49]. Regarding phase 2, the analysis of vascular function is evaluated at baseline and after 2 h from the intake of blueberry and control products. Also in this case, the choice to measure vascular function at 2 h derives from previous observations, performed both in our laboratories and in others, in which a significant increase in RHI was documented at that specific time from the intake of blueberries both in healthy subjects and in those with an endothelial dysfunction or other risk factors [30, 31, 45, 49]. The decision to split the blood samples collection and the analysis of vascular function into two different days was chosen to overcome the overlapping of the two procedures by reducing possible stress conditions to the volunteers, by facilitating the operating procedures and by limiting the occurrence of potential noises that could affect the different evaluations (vascular function and related markers).

As regards the primary outcome, RHI has been identified as a marker of vascular function that reflects endothelial function of the microvasculature (i.e., resistance vessels) [50]. RHI represents an alternative method to flow-mediated dilatation (FMD), the reference method, since it has the advantage of being simple, operator independent, and therefore less prone to operator bias and variability [36, 51]. In addition, RHI has shown a significant correlation (r = 0.55, p < 0.0001) with FMD [52, 53] and it is widely used, alone or in combination with FMD and/or other markers of vascular function, not only for clinical practice but also to study the effect of drugs, supplements, foods and exercise in numerous human interventional trials [54–58]. Regarding the secondary outcomes, a wide range of metabolic and functional markers related to the aging process, and directly and/or indirectly associated to vascular function, has been identified. In particular, glycaemia and insulin are analyzed in order to ascertain the impact of blueberries, as an important source of single sugars, on glucose metabolism and whether the eventual sugar metabolic response is able to influence vascular function. Finally, the analysis of markers of oxidative stress (e.g., DNA damage at cellular levels), inflammation and angiogenesis (e.g., interleukin-6, IL-6; tumor necrosis factor alpha, TNF-*α*; vascular endothelial growth factors; VEGF), adhesion molecules (vascular cell adhesion molecules 1, VCAM-1; intercellular adhesion molecules, ICAM-1), vasoconstrictors and vasodilators (ET-1 and NO, respectively) is useful to verify the contribution of blueberry in their modulation.

This study has some limitations. First, the difficulty of obtaining a real placebo, thus subjects are not blind to the treatment. Second, the relatively small sample size that, however, was defined based on previous studies obtained in different target groups [30, 31, 45] including older subjects [28]. It is noteworthy that the translation to the general older population could be limited since this target is characterized by wide heterogeneity, thus the demonstration in larger groups of volunteers could be recommended. Third, to limit exclusion criteria in order to enroll subjects with phenotypes that are typical of older age, including those presenting hypertension or mild pharmacological treatments even because in the cross over design adopted each subject will serve as their own control (i.e. also maintaining the same pharmacological treatments). In this regard, characteristics of subjects will be duly considered in data interpretation.

The results of the study will be able to provide several important pieces of information. First, to improve knowledge on the absorption of the bioactive compounds in older subjects, on which data are still lacking in literature. Second, to determine whether the intake of blueberries has a beneficial effect on vascular function also in relation to the absorption of its bioactives. Third, to verify the capacity of blueberries to positively modulate metabolic and functional markers, and whether their combination can provide evidence on the mechanisms by which the food/constituent could exert the protective effect. Overall, these results will be pivotal for the development of new dietary approaches in which blueberries and/or blueberry-products can be used, within a healthy dietary pattern, for improving vascular function with a view to promoting healthy aging.

## Supporting information

S1 Checklist SPIRIT 2013 checklist. (PDF)

S1 File Details regarding the study protocol. (PDF)

S2 File Ethical approval. (PDF)
