# Supplementary material for: Acute effect of blueberry intake on vascular function in older subjects: Study protocol for a randomized, controlled, crossover trial
Source: PLoS One. 2022 Dec 1;17(12):e0275132. doi: 10.1371/journal.pone.0275132 (PMC9714894; doi:10.1371/journal.pone.0275132)
Supplement: S1 File — (PDF) [file pone.0275132.s002.pdf]

S2 File. Ethical Approval

Prof. Patrizia Riso (Department of Department of Food, Environmental and Nutritional Sciences) informed the Ethics Committee of the University of Milan that she is going to conduct the following research: “Evaluation of the absorption kinetics of polyphenols from blueberry and role in the modulation of markers of vascular function and oxidative stress”.

The purpose of the study is to evaluate the absorption of some compounds of the class of polyphenols which are present in blueberries, and which are responsible for their blue-purple color (these compounds are in fact considered important for some protective activities).
The study also intends to evaluate the effect of blueberry intake on reduction of oxidative stress and improvement of vascular function which generally are critical factors during the aging process.

The Ethics Committee of the University of Milan examined and issued a favourable opinion on the project.

Milan, December 14th, 2020

IL PRESIDENTE
Margherita Ramajoli

Allegato 11 Comitato Etico 14.12.20
Repertorio pareri Comitato Etico: parere numero 121/20
