# Supplementary material for: Acute effect of blueberry intake on vascular function in older subjects: Study protocol for a randomized, controlled, crossover trial
Source: PLoS One. 2022 Dec 1;17(12):e0275132. doi: 10.1371/journal.pone.0275132 (PMC9714894; doi:10.1371/journal.pone.0275132)
Supplement: S2 File — (PDF) [file pone.0275132.s003.pdf]

S1 File. Details regarding the study protocol

STUDY 1

EVALUATION OF THE ABSORBPTION'S KINETICS OF BLUEBERRY POLYPHENOL AND THEIR ROLE IN THE MODULATION OF VASCULAR FUNCTION AND OXIDATIVE STRESS MARKERS

Introduction

Omissis

This trial aims to study the effect of the consumption of blueberries (selected for their high content of polyphenolic compounds) on the ability to modulate markers of endothelial function and oxidative stress in a group of elderly people. Since the protective effect seems to be dependent on the absorption of polyphenols, and that the latter may be different depending on the physiological characteristics of the subjects (e.g. age), it is very important to evaluate their absorption kinetics to better identify the plasma peak at which evaluate the function of the vascular endothelium. The results obtained from this study will be fundamental for the future development of dietary recommendations for specific targets, such as the elderly, who could benefit from the inclusion within their diet of foods that can improve the performance of the vascular system.

Recruitement

The study will be carried out on a group of healthy volunteers recruited, where possible, from the personnel belonging to the University of Milan, or through enrollment at the ICANS center (International Center for of Assessment of Nutritional Status) of DeFENS. The selection of the subjects will be carried out considering the general health conditions of the volunteers assessed through medical history and visits by the medical staff of ICANS. Inclusion criteria: men and women, age ≥ 60, absence of serious or disabling diseases. Exclusion criteria: subjects with allergies and/or intolerances

to the product and/or who declare that they have never consumed blueberries, use of supplements and/or dietary models and/or drugs with significant effects on the vascular function.
Volunteers will be invited to a meeting (if necessary, also remotely, using the web) in which the study will be illustrated in detail, given also the possibility for the volunteers to ask questions to clarify further doubts, as highlighted in the informative document.
At the end of this phase, the subjects can freely accept to participate as volunteers in the study and sign the informed consent that will be proposed to them.

Experimental design Test products

Blueberry belonging to the Legacy cultivar of the Vaccinium Corymbosum species will be used for the study, following the characterized by its composition in polyphenols and other bioactive compounds. This commercial product was obtained with organic cultivation and selected in the project for its high profile in polyphenolic compounds (anthocyanins in particular) with potential vasoactive properties. The product will be prepared on the day of experimentation starting from the frozen fruits that will be transformed into a homogeneous mousse before administration. Based on what was obtained in the previous intervention studies, a portion of 250 g was defined. The product will be taken by the volunteers in the morning. The control product will instead consist in a drink (250 mL) prepared with water and containing the same quantity and quality of sugars in blueberries, as already carried out in previous studies.

Study protocol

The study will be carried out according to a crossover, randomized, controlled experimental design. In detail, the subjects enrolled will be randomly allocated in one of the two intervention arms (test product vs control product), as shown in Figures 1 and 2, and will receive one of the two products according to the scheme; at the end of one of the two treatments (test arm or control arm), the groups will be reversed and whoever received the test product will receive the control and vice versa, at least one week apart. During the entire experimental period, the volunteers will be given dietary guidelines to follow.

PHASE I - STUDY OF BIOAVAILABILITY AND EFFECT ON MARKERS OF OXIDATIVE STRESS, INFLAMMATION AND VASCULAR FUNCTION

The enrolled subjects will be invited to maintain a low polyphenol content diet in the 24 hours preceding the experimentation. In this regard, the subjects will receive a list of products that they will have to exclude from their diet (for example drinks such as coffee, tea, red wine, and fruit juices) in the indicated period. On the day of the experiment, each subject will stay, for the period of the experiment, in the premises of the university structure, in a room specifically adhibited for study.

Volunteers will undergo a first venous sampling (t0, baseline) and anthropometric measurements (height, weight, and parameters of the state of nutrition). They will then be asked to consume the blueberry product or control and blood samples will be taken. The samples (6 mL) will be carried out at regular intervals of time (1h, 1.5 h, 2 h, and 4 h) to evaluate the absorption of polyphenols (e.g. anthocyanins) and other markers listed below. During the entire experimental period, the volunteers will be provided with dietary guidelines to follow. Blood samples will be taken by medical personnel from the ICANS, while the urine samples will be collected by the volunteers in sterile containers that will be delivered at the beginning and at the end of the experiment. The collected samples will then be delivered to the DeFENS research laboratory, treated, aliquoted and stored at -80 ° C until the time of analysis. From the blood samples we will proceed to the separation of plasma and/or serum, and lymphocytes for the evaluation of the different markers.

After a week the subjects will be called back to repeat the evaluations, administering the treatment not consumed during the first session (blueberry puree or the control product).

Analysis

Analyzes of polyphenols and salicylic acid, and their metabolites will be performed at plasma and / or urinary level by high performance liquid chromatography (HPLC-UV) and mass spectrometry (LC-MS) (Del Bo 'et al., 2012). The evaluation of DNA damage, as a marker of oxidative stress, will be performed at cell or whole blood samples by Comet assay.

The evaluation of vascular function markers (e.g. nitric oxide, endothelin-1, VEGF, VCAM-1, ICAM-1) and inflammation (e.g. IL-6, IL-8, TNF-α) will be evaluated at serum through the use of ELISA kits.

Fig. 1 Experimental design of the bioavailability study and effect on oxidative stress, vascular function, and inflammation.

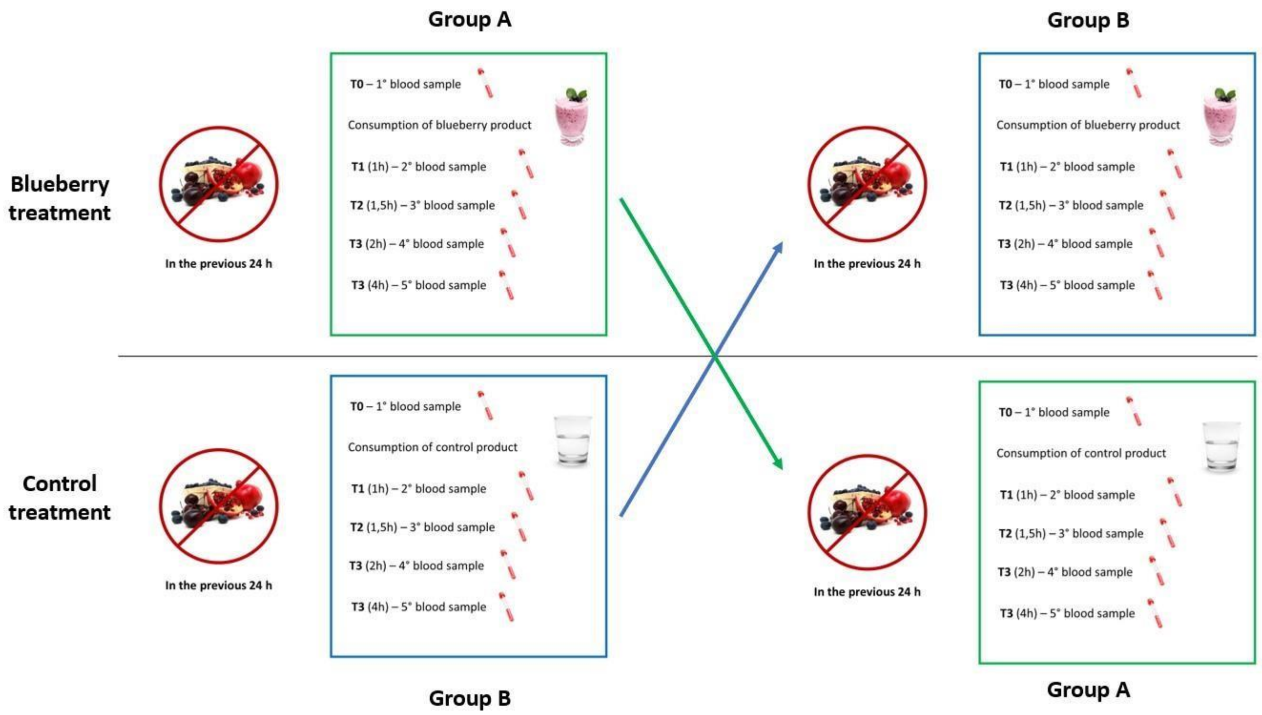

PHASE II - ANALYSIS OF ENDOTHELIAL FUNCTION BY USING BIOSENSORS

The subjects involved in phase I, after at least one week, will be invited to return for the evaluation of the endothelial function carried out using the Endo-PAT2000 biosensor method. This instrument uses a non-invasive plethysmographic method that measures the pulsatile blood volume of the small arteries at the level of the fingertips of both hands and provides an estimate of two indices: the Reactive Hyperemia Index (RHI, which measures NO-dependent vascular changes and the transient increase in blood flow following a short occlusion) and the Augmentation Index (AI, marker of arterial stiffness), both related to vascular function and also widely used in studies to evaluate the modulatory efficacy of small fruits (eg. blueberries, strawberries, blackberries, raspberries).

On the day of the experiment, the volunteers will stay on the premises of the university structure in a room specially dedicated to them. The subjects will undergo an initial evaluation of vascular function (t0, baseline) and pressure. They will then be asked to consume the blueberry product or control and will then undergo the second vascular function assessment. This evaluation will be carried out in correspondence with the anthocyanin absorption peak (results from phase 1), which based on previous studies should correspond to approximately 2 hours after taking the product (Figure 2). After

a week the subjects will be called back to repeat the evaluations, administering the treatment not consumed during the first session (blueberry puree or the control product).

Fig. 2 Experimental design of the analysis of the vascular function by biosensors.

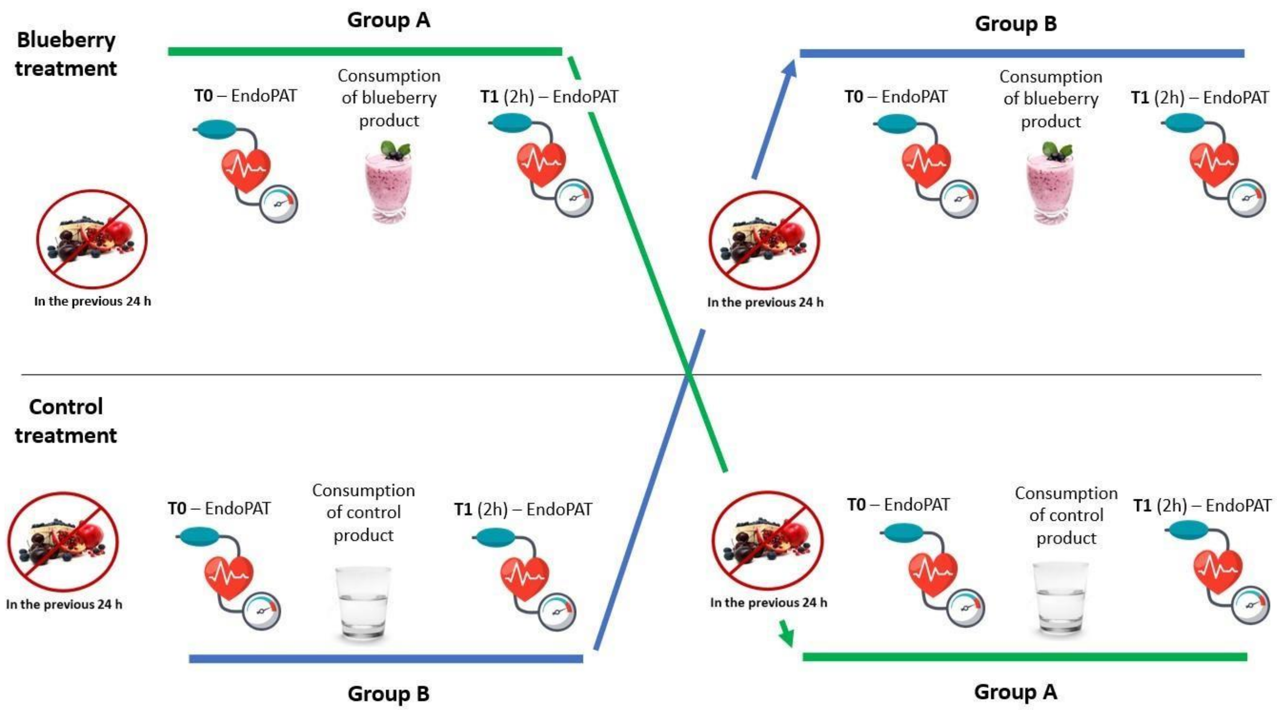

Analysis of markers

The markers being assessed are the following:

-RHI and FRHI (as indices of reactive hyperemia), AI and AI @ 75 (arterial stiffness indices). These values will be obtained directly through the processing of the EndoPAT2000 device

- systolic and diastolic pressure assessed by using the sphygmomanometer following standard procedures.

STATISTICAL DATA ANALYSIS

28 volunteers will be enrolled for the study. This number was considered sufficient (alpha = 0.05, 80% of potency), based on previous studies, to determine an increase of 0.30 in the RHI parameter (endothelial function marker) after blueberry intake. This calculation also considers a possible drop out of up to 30% of the volunteers.

The statistical analysis of the data will be performed in general by verifying the distribution of the data and applying the two-way ANOVA for repeated measurements considering the treatment factor (food test vs. control) and the time factor as dependent variables. A p value <0.05 will be considered statistically significant. Analyzes will also be planned to verify any correlations between the various markers analyzed.
